# Effect of prophylactic 360° laser treatment for prevention of retinal detachment after phacovitrectomy: (Prophylactic 360° laser treatment for prevention of retinal detachment)

**DOI:** 10.1186/1471-2415-13-77

**Published:** 2013-12-10

**Authors:** Takeshi Iwase, Young-Joon Jo, Brian C Oveson

**Affiliations:** 1 Department of Ophthalmology, Nagoya University Hospital, Nagoya, Japan; 2 Department of Ophthalmology, Toyama Prefectural Central Hospital, Toyama, Japan; 3 Department of Ophthalmology, Johns Hopkins University School of Medicine, Baltimore, MD, USA; 4 Department of Ophthalmology, Chungnam National University Hospital, Daejeon, Korea; 5 State University of New York at Stony Brook University Hospital and Medical Center, Stony Brook, NY, USA

**Keywords:** Phacovitrectomy, Prophylactic 360° laser, Retinal detachment, Macular hole

## Abstract

**Background:**

To investigate the effect of intraoperative 360° laser retinopexy anterior to the equator for the prevention of retinal detachment after phacovitrectomy.

**Methods:**

The patients were part of two consecutive case series cohorts in macular hole (MH) and rhegmatogenous retinal detachment (RRD), one which did not receive intraoperative prophylactic 360° laser, and one which received intraoperative prophylactic 360° laser. For the 360° laser treatment group, three rows of medium-white burns were positioned anterior to the equator. The baseline characteristics and the risk of retinal detachment over time were analyzed and compared between the groups.

**Results:**

Prophylactic intraoperative 360° laser treatment was performed on 77 MH cases (67.3 years) and compared to a control group of 35 MH cases (65.8 years). Additionally, prophylactic intraoperative 360° laser treatment was performed on 108 RRD cases (64.0 years) and compared to 270 RRD cases (64.4 years). The 360° laser group showed a significant reduction (0%, 0/77 eyes) in the rate of the incidence of retinal detachment after vitrectomy at 12 months after surgery in MH cases, compared with the control group (5.7%, 2/35 eyes) (p = 0.034). Kaplan-Meier survival analysis demonstrated that the rate of retinal detachment in the control group was significantly higher than that in the 360° laser group (p = 0.035). There was no significant difference between the groups in RRD cases (p = 0.092).

**Conclusions:**

Intraoperative 360° laser retinopexy following phacovitrectomy resulted in a significant reduction in the rate of postoperative retinal detachment in MH cases.

## Background

Retinal detachment is one of the most severe complications following vitrectomy. The incidence of retinal detachment after vitrectomy is 6–8% in epi-retinal membrane (ERM) cases [1,2], 6% in branch retinal vein occlusion cases [3], and varies between 0-11% in macular hole (MH) cases [4-9]. Retinal detachment may be attributable to the surgical technique, and can be affected by how much of the peripheral vitreous remained in MH cases. The presence of a gas bubble in an incompletely vitrectomized eye can induce traction on the peripheral retina, leading to formation of peripheral breaks and retinal detachment. Due to the vision-threatening nature of this complication, many attempts have been made to prevent retinal detachment, including; careful peripheral retinal examination with scleral depression, properly treating retinal breaks at the end of the vitrectomy, and even prophylactic scleral buckling [10] or cryopexy [11]. Combined vitrectomy and phacoemulsification (phacovitrectomy) surgery has several advantages that prevent retinal re-detachment. Phacoemulsification surgery results in; a more complete vitrectomy, the possibility of a more efficient scleral depression, and an entry site that is more anterior, thus, reducing the risk of entry site complications. However, in our experience, even with the many advantages of the combined phacovitrectomy surgery, retinal detachment following surgery could not be completely prevented. Therefore, it would be beneficial if an alternative, less-invasive management strategy was feasible. The incidence of retinal re-detachment was reduced by 50% with prophylactic 360° laser retinopexy after removal of silicone oil [12,13]. We hypothesize that 360° laser retinopexy anterior to the equator can reduce the incidence of retinal breaks and detachment by causing strong chorioretinal adhesion. It may also prevent the progression of retinal detachment similar to demarcation laser treatment. This procedure can easily be performed during the vitrectomy procedure with the endolaser probe. The purpose of this study was to evaluate the effect of intraoperative 360° prophylactic laser retinopexy on the incidence of retinal detachment after phacovitrectomy using a case-control design in MH and rhegmatogenous retinal detachment (RRD) cases.

## Methods

We retrospectively reviewed the patient records to identify patients who underwent phacovitrectomy surgery for idiopathic MH and RRD by a single surgeon (T.I.) in Toyama Prefectural Central Hospital between July 2000 and September 2007.

The patients were part of two consecutive case series cohorts in each diagnosis, one which received intraoperative prophylactic 360° laser (June 2001-December 2004), and one which did not receive intraoperative prophylactic 360° laser (January 2005-September 2007). Surgery was carried out based on the approval of the institutional review board in Toyama Prefectural Central Hospital and the ethical standard established by the Declaration of Helsinki. After an explanation, informed consent was obtained from all patients.

Each patient underwent a detailed preoperative evaluation. The following patient information was also collected: age, gender, systemic disease, previous ocular surgery, and associated eye diseases. Postoperatively, a complete ocular examination was performed on the 1, 3, 7 days, 2, 4, 6, 8, 10 weeks, and every month until 12 months after surgery. All patients were followed for twelve months after surgery.

Exclusion criteria were; previous ocular surgery, giant tears, retinal dialysis, trauma, proliferative vitreoretinopathy (PVR, grade C or higher), retinal detachment with macular hole (high myopia), or round hole detachment with no associated PVD.

### Surgical technique

After administration of retrobulbar and peribulbar anesthesia (2% lidocaine hydrochloride), a half-round fornix-based conjunctival incision was created. Conventional 20-gauge PPV was conducted using an Accurus 800CS (Alcon Inc., Fort Worth, TX).

First, cataract surgery was performed as described below. After a 3.0 mm wide self-sealing superior sclerocorneal tunnel was created at 12 o’clock, the continuous curvilinear capsulorhexis was made. The nucleus was removed using the phaco-chop method and the residual cortex was aspirated by an irrigation/aspiration (I/A) tip. Next, a foldable acrylic IOL SA60AT (Alcon Inc.) was implanted into the bag.

The sclerotomies were performed 3.5 mm from the limbus. Triamcinolone acetonide was used intraoperatively to highlight the vitreous body, and the posterior cortical vitreous up to the vortex vein was removed. ICG staining was employed for MH patients, and the ILM was removed at least 2 disc diameters from the MH edge. The eyes underwent 360° scleral depression to trim the vitreous in the vitreous base and confirm an absence of iatrogenic retinal breaks. If a retinal break was found during the surgical procedure, it was treated with endo-laser for both the treatment and the control groups. After fluid-air or gas (20% SF_6_) exchange, the original retinal break was treated with endo-laser. The 360° laser retinopexy involved placement of three rows of medium-white burns anteriorly from the level of vortex vein, towards and beyond the equator with burns approximately one burn width apart using the endo-laser system. The sclerotomies were sutured with 7-0 Vicryl.

Following MH surgery, patients were instructed to remain in a face-down position for 12 hours per day for a minimum of 1 week. The RRD patients were not instructed to lay face-down following surgery.

### Visual acuity

Best-corrected visual acuity on decimal charts was recorded at each visit and this acuity was converted to the logarithm of the minimal angle of resolution (logMAR) for statistical analysis.

### Statistical analysis

Baseline demographic and clinical characteristics were examined using unpaired *t*-test or Fisher exact test. The time to retinal detachment after vitrectomy was analyzed using the Kaplan-Meier product-limit method and log rank test. Continuous variables without a normal distribution were compared using the Mann-Whitney *U* test. Differences with a p-value less than 0.05 were considered statistically significant.

## Results

### Baseline characteristics of study cohort

The study included 112 consecutive MH cases and 378 RRD cases. Demographics such as age, gender, macula status (in RRD), preoperative vision, axial length, and type of tamponade are summarized in Table 1. There was no statistically significant difference in baseline characteristics between the groups in MH and RRD cases.

**Table 1:** Patients characteristic

		**Macular hole**			**Retinal detachment**	
	**360° Laser group**	**Control group**	**P**	**360° Laser group**	**Control group**	**P**
Number	77	35	-	108	270	-
Age(year)	67.3 ± 5.7	65.8 ± 8.8	0.360	64.0 ± 11.5	64.4 ± 10.8	0.756
Gender(M/F)	32/35	12/23	0.192	60/48	138/132	0.434
Macula status (On/Off)	-	-	-	72/36	166/104	0.346
Pre-operative VA	20/95	20/85	0.487	20/85	20/78	0.688
Axial length(mm)	23.8 ± 1.4	23.6 ± 1.5	0.495	24.4 ± 2.4	24.7 ± 2.8	0.297
Vitreous hemorrhage(eye)	0	0	1	7	31	0.144
Retinal break(single/multiple)	-	-	-	30/78	84/186	0.523
Retinal break(Sup/Inf/both sides)	-	-	-	65/14/29	189/32/49	0.134
Retinal break(tear(s)/hole(s))	-	-	-	84/24	194/76	0.238
Lattice degeneration(+/-)	-	-	-	23/85	61/209	0.784
Tamponade(Air/SF_6_)	0/77	0/35	1	75/33	188/69	0.972

Sup: superior, Inf: inferior.

Prophylactic intraoperative 360° laser treatment was performed on 77 consecutive MH cases (68.8%, mean age of 67.3 years) and compared to a control group of 35 consecutive MH cases (31.2%, mean age of 65.8). Prophylactic intraoperative 360° laser treatment was performed on 108 consecutive RRD cases (28.5%, mean age of 64.0 years) and compared to a control group of 270 consecutive RRD cases (61.5%, mean age of 64.4).

### Incidence of retinal detachment

**Figure 1:**
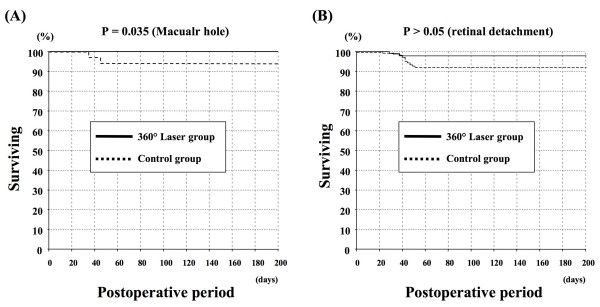
**Kaplan-Meier survival curves of the treatment group and the control group, showing the cumulative proportion of retinal detachment at various time intervals. (A)** There was a statistically significant difference between the two curves in the macular hole cases (p = 0.035). **(B)** There was no statistically significant difference between the two curves in rhegmatogenous retinal detachment cases (p > 0.05).

The 360° laser group showed a significant reduction (0%, 0/77 eyes) in the rate of the incidence of retinal re-detachment after vitrectomy at 12 months following surgery for the MH cases, compared with the control group (5.7%, 2/35 eyes) (p = 0.034) (Table 2). One eye had a new retinal break that corresponded to the sclerotomy site, and the other eye had a new retinal break at the inferonasal quadrant, which was not related to the screlotomy. Kaplan-Meier survival analysis demonstrated that the rate of retinal detachment in the control group was significantly higher than that in the 360° laser group (p = 0.035, Figure 1). Postoperative retinal detachment was 5.9% of the control group (3 eyes had a new retinal break not correlated to the sclerotomy, and 13 eyes had RD with re-opening of the causative retinal break(s) because of proliferative vitreoretinopathy) and 1.9% of the 360° laser group in RRD cases (2 eyes had RD with re-opening of the causative retinal break(s) because of proliferative vitreoretinopathy. Notably, the location on the retina where the 360° laser retinopexy was performed, was not re-detached). However, there was no significant difference between these groups (p = 0.092). None of the patients developed retinal re-detachment in any of the groups beyond 2 month after surgery. Kaplan-Meier survival analysis demonstrated that there was no significant difference between the groups in RRD cases (p > 0.05) (Figure 1).

**Table 2:** Complications

		**Macular hole**			**Retinal detachment**	
	**360° Laser group**	**Control group**	**P**	**360° Laser group**	**Control group**	**P**
Retinal detachment	0(0%)	2(5.7%)	0.034	2(1.9%)	16(5.9%)	0.092
Epiretinal membrane	0(0%)	0(0%)	1	7(6.5%)	10(3.7%)	0.239
Macular hole	-	-	-	0(0%)	3(1.1%)	0.274
(Initial failure)	3(3.9%)	1(2.9%	0.784	-	-	-
(Final failure)	0(0%)	0(0%)	1	-	-	-
Cystoid macular edema	0(0%)	0(0%)	1	1(0.9%)	6(2.2%)	0.669
Vitreous hemorrhage	0(0%)	0(0%)	1	1(0.9%)	2(0.7%)	0.855

### Other complications

The proportions of ERM, development of MH in RRD cases, failure of MH in MH cases, cystoid macular edema (CME), and vitreous hemorrhage were not statistically different between the two groups (Table 2). Two eyes had rupture of the posterior capsule as a complication of cataract surgery in this study, but had no postoperative complication including retinal detachment after surgery.

Postoperative ERM developed in 7 eyes (6.5%) from the 360° laser group and in 10 eyes (3.7%) from the control group in the RRD cases. Within the MH cases, neither group developed ERM. The initial failure of MH rate was 3 (3.9%) in the 360° laser group, and 1 (2.9%) in the control group. The final failure rate was 0% in both groups. Postoperative MH developed in 3 (1.1%) patients within the RRD control group.

CME was observed in 1 eye (0.9%) from the 360° laser group and 6 eyes (2.2%) from the control group in RRD cases.

Vitreous hemorrhage occurred 1 eye (0.9%) from the 360° laser group and 2 eyes (0.7%) from the control group in RRD cases. There was no statistically significant difference within the RRD groups. There was no development of vitreous hemorrhage in any of the MH cases.

### Visual acuity

There was no statistically significant difference in postoperative visual acuity (log MAR; the 360 laser group: 0.19 ± 0.26 compared to the control group: 0.28 ± 0.28, P = 0.112) in MH cases and (log MAR; the 360° laser group: 0.11 ± 0.26 compared to the control group: 0.07 ± 0.29, P = 0.193) in RRD cases (Table 3).

**Table 3:** Visual acuity

		**Macular hole**			**Retinal detachment**	
	**360° Laser group**	**Control group**	**P**	**360° Laser group**	**Control group**	**P**
Preoperative VA	20/95	20/85		20/85	20/78	
(Log MAR)	0.68 ± 0.33	0.63 ± 0.36	0.487	0.63 ± 0.86	0.59 ± 0.91	0.688
Postoperative VA	20/31	20/37		20/27	20/23	
(Log MAR)	0.19 ± 0.26	0.28 ± 0.28	0.112	0.11 ±0.26	0.07 ± 0.29	0.193

## Discussion

Iatrogenic retinal breaks and retinal detachment remain a severe vision-threatening complication following vitreous surgery. There are two main causes of iatrogenic retinal breaks associated with vitrectomy. One is that insertion of an instrument may cause traction on the adjacent vitreous resulting in a retinal tear along the posterior border of the vitreous base intraoperatively. Additionally, the vitreous may become immobilized within the sclerotomy site during withdrawal of an instrument causing traction and a retinal break along the posterior border of the vitreous base postoperatively. Careful peripheral retinal examination with scleral depression should detect breaks induced by this manner, and appropriate retinopexy may prevent the development of subsequent retinal detachment.

Theocharis et al. noted that phacovitrectomy leads to a more complete vitrectomy, the possibility of a more efficient scleral depression, and a more anterior entry site placement with a reduced risk of entry site complications [14]. Accordingly, the phacovitrectomy has several advantages to prevent retinal detachment after surgery. In our study, cataract surgery was performed by phacoemulsification prior to vitrectomy. However, the control group contained 16 (5.9%) patients with retinal detachments within RRD cases, and 2 (5.7%) retinal detachments in MH cases after surgery in the present study. In the RRD cases, a single-operation without scleral buckling anatomic success rate was reported between 62 and 100% in pseudophakic patients [15-23] and between 64–80% in phakic patients [16,17]. In MH cases, Sjaarda et al., found incidence of RD in 1.1% and retinal breaks in 5.5% [5], while Park et al., reported a significantly higher rate of RD (14%) and peripheral retinal tears (3%) [24]. The vitrectomy results for the Macular Hole Study Group found a comparably high incidence of retinal detachment (11%) [4]. Even though the rate of retinal re-detachment after surgery was not higher in our study compared to the previous reports, a method to reduce complications would be beneficial.

To minimize the incidence of postoperative retinal detachment, prophylactic measures have been proposed. Scleral buckling [10] or circular cryoretinopexy [11] were deemed appropriate prophylactic measures but these are in fact quite invasive and impose risks of their own. On the other hand, intraoperative 360° laser during vitrectomy can treat unseen breaks or prevent formation of new breaks and prevent retinal detachment after vitrectomy. This procedure takes only a few minutes to complete, and can be performed while observing the peripheral retina during depression of the sclera. There is mounting evidence that shows the advantages of 360° laser retinopexy after SO removal [12,13]. In a wide retrospective study in patients with RRD, RD after oil removal occurred in 26% vs 14% of laser-treated eyes (p = 0.01) [12].

In eyes with vitreal or macular diseases, excluding RD which underwent vitrectomy, Koh et al., found that intraoperative 360° laser retinopexy was associated with a reduction in the incidence of RD after surgery from 13.3% to 3.5% [25]. However, most of their cases did not have fluid-air/gas exchange nor any tamponade. Using the tamponade at the end of the vitrectomy allows sufficient time for chorioretinal adhesion to develop [26,27]. Therefore, the advantages of our study is that the effect of the 360° laser retinopexy is compared in the same diagnosis and the tamponade was used for each case.

In our study, intraoperative 360° laser during vitrectomy resulted in no retinal detachment after surgery and a significant reduction in the rate of postoperative retinal detachment in MH cases. Furthermore, regarding complications, the initial failure of MH closure was not significantly different between the groups, and complete closure occurred in all of the MH cases. Also there was no significant difference between the groups regarding other complications in our MH cases. The results indicate that the present procedure is useful for preventing retinal detachment after vitrectomy.

Although the rate of retinal re-detachment was lower in the 360° laser group, there was no significant difference between the 360° laser group and the control group in RRD cases. The cause of retinal re-detachment after vitrectomy is probably multifactorial, and is mainly dependent on the presence of two preconditions; inflammation and responsive cells [28]. The logical goal of a vitrectomy procedure in RRD would be to remove such cells and also their substrates of attachment (vitreous collagen) without causing an increased inflammatory response. However, this is further complicated by the fact that the use of vitrectomy combined with laser photocoagulation has been identified as a risk factor for PVR development [29,30]. In previous studies, the degree of vitreous removal ranges from a core vitrectomy followed by removal of the vitreous adherent to breaks [31], to a complete 360° shaving of the vitreous base [18,23]. Intraoperative 360° laser, as well as cryopexy, might cause the breakdown of the blood–retinal barrier with leakage of serum proteins into the intraocular fluids [32]. This could be the source of cellular migration and proliferation resulting in epiretinal membrane formation. In our study, the occurrence of ERM was not significantly different between the groups. Taken together, the 360° laser procedure for RRD is still controversial. Another possible method of resolution is the use of cannulated vitrectomy systems. These systems may protect the vitreous base by allowing easier entry of instruments and cause less frequent herniations of the vitreous into the scleral incision [33].

In this study, 20-G vitrectomy was performed in all of the cases. Recently, 23- or 25- G vitrectomy may be preferable, because the smaller gauge vitrectomy has several advantages. The incidence of retinal breaks in 20-G vitrectomy in MH surgery was found to be between 0% and 7.2% [34-36]. In 23- or 25-gauge vitrectomy incidences were reported to be 0% to 3.1% [6,7,37,38]. Using smaller gauge instruments might decrease trauma in and near the vitreous base adjacent to the sclerotomies. Furthermore, the use of trocars might protect the vitreous base from excessive traction to the adjacent retina. Although the incidence of retinal breaks has decreased in small gauge vitrectomy, the possibility of postoperative retinal detachment has not been eliminated. The presence of a gas bubble can induce traction on the peripheral retina, an inflammation reaction, or vitreoretinal proliferation development, leading to formation of peripheral breaks and retinal detachment. These results suggest that 360° laser treatment may be an option for preventing retinal detachment postoperatively in vitrectomy. Smaller gauge instruments would further decrease the incidence of complications.

## Conclusions

A more complete vitrectomy with the intention of reducing the amount of residual peripheral vitreous with scleral indentation, and a careful sclerotomy cleansing of incarcerated vitreous should be performed at the end of surgery. However, it is impossible to completely prevent postoperative retinal detachment caused by unseen retinal breaks during surgery or postoperatively. Considering the vision-threatening nature of retinal re-detachment after vitrectomy, this adjunctive treatment should be considered during vitrectomy procedures for the prevention of postoperative retinal detachment. A randomized, prospective clinical trial is necessary to definitively determine the efficacy of this treatment.

### Ethical approval

Surgery was carried out based on the approval of the institutional review board and the ethical standard established by the Declaration of Helsinki. An informed consent was obtained from all of the patients.

## Competing interests

None of the authors have a financial or proprietary interest in any product mentioned.

## Authors’ contribution

TI made a substantial contribution to conception and design, analysis and interpretation of data, drafting the manuscript and revising it critically for important intellectual content. YJ and BO were involved in study conception and design and analysis and interpretation of data. All authors read and approved the final manuscript.

## Pre-publication history

The pre-publication history for this paper can be accessed here:

http://www.biomedcentral.com/1471-2415/13/77/prepub
